# Peroxisome Proliferator-Activated Receptor γ Is a Target for Halogenated Analogs of Bisphenol A

**DOI:** 10.1289/ehp.1003328

**Published:** 2011-05-11

**Authors:** Anne Riu, Marina Grimaldi, Albane le Maire, Gilbert Bey, Kevin Phillips, Abdelhay Boulahtouf, Elisabeth Perdu, Daniel Zalko, William Bourguet, Patrick Balaguer

**Affiliations:** 1 INRA (National Institute of Agronomic Research), UMR 1089 Xénobiotiques, Toulouse, France; 2 IRCM(Institut de Recherche en Cancérologie de Montpellier), Montpellier, France; 3 INSERM (Institut National de la Santé et de la Recherche Médicale) U896, Montpellier, France; 4 Université Montpellier 1, Montpellier, France; 5 CRLC (Centre Régional de Lutte contre le Cancer) Val d’Aurelle Paul Lamarque, Montpellier, France; 6 INSERM U1054, Centre de Biochimie Structurale, Montpellier, France; 7 CNRS (Centre National de la Recherche Scientifique) UMR5048, Universités Montpellier 1 & 2, Montpellier, France; 8 NovAliX, Illkirch, France; 9 Methodist Hospital Research Institute, Houston, Texas, USA

**Keywords:** BPA, endocrine disruptor, obesity, PPARγ, TBBPA, TCBPA

## Abstract

Background: The occurrence of halogenated analogs of the xenoestrogen bisphenol A (BPA) has been recently demonstrated both in environmental and human samples. These analogs include brominated [e.g., tetrabromobisphenol A (TBBPA)] and chlorinated [e.g., tetrachlorobisphenol A (TCBPA)] bisphenols, which are both flame retardants. Because of their structural homology with BPA, such chemicals are candidate endocrine disruptors. However, their possible target(s) within the nuclear hormone receptor superfamily has remained unknown.

Objectives: We investigated whether BPA and its halogenated analogs could be ligands of estrogen receptors (ERs) and peroxisome proliferator–activated receptors (PPARs) and act as endocrine-disrupting chemicals.

Methods: We studied the activity of compounds using reporter cell lines expressing ERs and PPARs. We measured the binding affinities to PPARγ by competitive binding assays with [^3^H]-rosiglitazone and investigated the impact of TBBPA and TCBPA on adipocyte differentiation using NIH3T3-L1 cells. Finally, we determined the binding mode of halogenated BPAs to PPARγ by X-ray crystallography.

Results: We observed that TBBPA and TCBPA are human, zebrafish, and *Xenopus* PPARγ ligands and determined the mechanism by which these chemicals bind to and activate PPARγ. We also found evidence that activation of ERα, ERβ, and PPARγ depends on the degree of halogenation in BPA analogs. We observed that the bulkier brominated BPA analogs, the greater their capability to activate PPARγ and the weaker their estrogenic potential.

Conclusions: Our results strongly suggest that polyhalogenated bisphenols could function as obesogens by acting as agonists to disrupt physiological functions regulated by human or animal PPARγ.

Bisphenols form a large family of chemicals that are used mainly to produce polycarbonates and epoxy resins. By far, the most widely used bisphenol (> 3 million tons/year) is bisphenol A (BPA), which is used in the manufacture of items such as plastics, food can linings, dentistry sealants, and thermal paper. BPA (Figure 1) is a model xenoestrogen. Despite possessing only modest estrogenic activity compared with 17β-estradiol (E2), over the last decade BPA has been shown to produce a range of adverse effects in laboratory animals, with major concerns regarding reproductive targets (Richter et al. 2007). More recently, it has been hypothesized that early exposure to BPA could play a role in the onset of obesity and other metabolic syndromes (Rubin and Soto 2009). In this regard, a large body of data about endocrine-disrupting chemicals (EDCs) underlines the importance of exposure during early stages of development, which could result in reproductive defects in adult life (Newbold et al. 2009). Human exposure to BPA has been clearly demonstrated (Calafat et al. 2008). However low-dose effects of BPA and the possible consequences of such exposure are controversial (Vandenberg et al. 2009; vom Saal and Hughes 2005).

**Figure 1:**
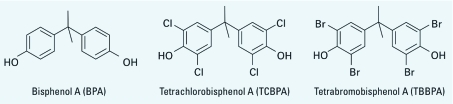
Chemical structures of BPA, TBBPA, and TCBPA.

Halogenated derivatives of BPA, which feature bromine or chlorine substituents on the phenolic rings, are used as flame retardants. However, compared with BPA, little information is available regarding the potential endocrine disruption by these compounds. All brominated BPA analogs originate from tetrabromobisphenol A (TBBPA), which is the most-produced brominated flame retardant (> 150,000 tons produced annually) (de Wit et al. 2010). TBBPA (Figure 1) is used to produce fireproof epoxy resins used in the manufacture of computer motherboards and other electronics; and it has been found in the environment (de Wit et al. 2010), in wildlife (Darnerud 2003), and in human samples (Cariou et al. 2008; Shi et al. 2009). TBBPA is debrominated in the environment into lower-brominated BPA analogs (monoBBPA, diBBPA, and triBBPA) (Arbeli et al. 2006). The closely related tetrachlorobisphenol A (TCBPA) (Figure 1) is also used as a flame retardant, but in much lower quantities than TBBPA (< 10 000 tons/year) (Chu et al. 2005), and its presence in environmental samples has been unequivocally demonstrated (Fukazawa et al. 2001). Given the low production level of TCBPA, its presence in the environment most likely originates from the spontaneous chlorination of BPA. Indeed, like many phenolic compounds, BPA is readily chlorinated in aqueous media (Deborde et al. 2004).

The estrogenic activity of BPA exerted through binding to estrogen receptors (ERs) is likely involved in the onset of many of its adverse effects, and several studies in animal models have shown that such effects are observed after exposure to low doses (Vandenberg et al. 2009). Brominated BPA analogs are not as estrogenic as BPA, and the potency of brominated-BPAs as ER agonists decreases as the number of bromine atoms increases (Meerts et al. 2001). Conversely, the estrogenic activity of chlorinated congeners could be similar to or higher than that of BPA (Mutou et al. 2006; Takemura et al. 2005). Similarly, both TBBPA and TCBPA interact with and disrupt thyroid hormone receptor signaling (Kitamura et al. 2002). Recently, Somm et al. (2009) showed that perinatal exposure to BPA altered early adipogenesis in the rat, which is mediated by peroxisome proliferator–activated receptor γ (PPARγ), a nuclear hormone receptor whose dysregulation is involved in the onset of diabetes and obesity (Swedenborg et al. 2009). This suggests that BPA and its derivatives may also interact with this receptor. In the present study, we examined the capacity of BPA and halogenated BPA derivatives to interact with and perturb signaling by ERα, ERβ, PPARα, PPARδ, and PPARγ. We provide the first experimental evidence that flame retardants TBBPA and TCBPA are ligands and partial agonists of human PPARγ and also activate the corresponding zebrafish and *Xenopus* receptors. Our findings indicate that these compounds should certainly be evaluated as EDCs with possible deleterious effects on humans and wildlife.

## Material and Methods

*Chemicals.* We purchased E2, perfluorooctanesulfonic acid (PFOS), perfluorooctanoic acid (PFOA), BPA, and TBBPA [2,2-bis(3,5-dibromo-4-hydroxyphenyl)propane] from Sigma-Aldrich (Saint-Quentin Fallavier, France); TCBPA [2,2-bis(3,5- dichloro-4-hydroxyphenyl)propane] from TCI Europe (Zwijndrecht, Belgium); and rosiglitazone from Interchim (Montluçon, France). Mono-2-ethylhexyl phthalate (MEHP) was a gift from M.C. Chagnon (AgroSup Dijon, Dijon, France). [^3^H]-Rosiglitazone (2,000 GBq/mmol) was purchased from PerkinElmer (Courtaboeuf, France). We obtained materials for cell culture from Invitrogen (Cergy-Pontoise, France) and luciferin from Promega (Charbonnieres, France). 3-MonobromoBPA, 3,3´-dibromoBPA, and 3,3´,5-tribromoBPA were synthesized from BPA. Brominated-BPA analogs were individually isolated and purified using HPLC and a Gilson 202 fraction collector (Gilson France, Villiers-le-bel, France) as described previously (Zalko et al. 2006). We evaluated the purity of all brominated analogs by ultraviolet HPLC, electrospray ionization mass spectrometry, and nuclear magnetic resonance (> 99.8%).

*Transient transfection experiments.* We monitored human (h), zebrafish, and *Xenopus* (x) PPARγ activity on (GAL4RE)_5_-βglobin-luciferase and (PPRE)_3_-TK-luciferase reporter constructs. PSG5-GAL4-hPPARγ-puro, pSG5-GAL4-puro, (GAL4RE)_5_-β-glob-luciferase, (PPRE)_3_-TK-luciferase were tested previously (le Maire et al. 2009). PSG5-xPPARγ was a gift from W. Wahli (University of Lausanne, Lausanne, Switzerland). Zebrafish PPARγ ligand-binding domain (LBD) was synthesized by Eurofins MWG Operon (Les Ulis, France) and cloned between *Bam*HI and *Xho*I restriction sites in pSG5-GAL4-puro. Transient transfection and luciferase assays were performed as previously described (le Maire et al. 2009).

*Reporter cell lines and stable gene expression assay.* Generation of HGELN, HGELN-ERα, HGELN-ERβ, HGELN-GAL-PPARα, HGELN-GAL-PPARβ, and HGELN-GAL-PPARγ reporter cell lines was performed as previously described (Escande et al. 2006; le Maire et al. 2009). Briefly, reporter cells were seeded at a density of 20,000 cells/well in 96-well white opaque tissue culture plates and maintained in phenol-red–free Dulbecco’s modiﬁed Eagle’s medium (DMEM) supplemented with 5% dextran-coated, charcoal-treated fetal calf serum. Twenty-four hours later, culture medium was replaced with DMEM containing tested compounds. We performed assays in the absence of serum to avoid ligand capture by serum proteins. Sixteen hours after exposure, we replaced media with media containing 0.3 mM luciferin. Luminescence was measured in intact living cells for 2 sec in a Microbeta Wallac luminometer (PerkinElmer).

*NIH3T3-L1 differentiation.* Two-day postconfluent 3T3L1 preadipocytes (a gift from L. Fajas; Institut de Génétique Moléculaire, Montpellier, France) were induced to differentiate by 2-day treatment with a differentiation mixture (10 μg/mL insulin, 1 μM dexamethasone, 0.5 mM isobutylmethylxanthine) followed by 8-day treatment with 10 μg/mL insulin and PPARγ ligands. The medium was replaced every 48 hr. After differentiation, cells were stained with Oil Red O for morphological analyses, or RNA was extracted from the cells using the RNeasy RNA isolation kit (Qiagen, Courtaboeuf, France). For RNA extractions, four independent cultures were performed per condition. Reverse transcription was performed with random hexamers on 1 μg total RNA using SuperScript II reverse transcriptase (Invitrogen), and the reaction was diluted 100 times for amplification. Real-time polymerase chain reaction (PCR) quantification was then performed using SYBR Green technology (LightCycler; Roche Diagnostics, Meylan, France). Results were normalized to two housekeeping genes (18S and 36B4) and quantified using qBase (Roche Diagnostics).

*PPAR*γ *expression and purification.* DNA encoding the LBD of human PPARγ (amino acids Glu196-Tyr477) was amplified by PCR and cloned into the expression vector pET15b. The plasmid PPARγ (Glu196-Tyr477)-pET15b was transformed into *Escherichia coli* BL21(DE3) cells (Invitrogen). The PPARγ LBD was expressed and purified as previously described for retinoid X receptor (RXR)α LBD (Nahoum et al. 2007). Prior to crystallization trials, the purified PPARγ LBD was concentrated to 8.5 mg/mL in a buffer containing 20 mM Tris-HCl, pH 8.5, 250 mM NaCl, 5 mM dithiothreitol, and 1 mM EDTA.

*Crystallization.* Crystals were obtained by vapor diffusion in hanging drops at 293 K. For crystals of unliganded (apo) PPARγ, 1 µL protein solution was mixed with 1 µL well solution containing 1 M trisodium citrate, pH 7.5, 100 mM Hepes, pH 7.5, and 3% 1,2-propanediol. Crystals appeared after 1 day and grew to about 200 µm within a few days. TCBPA was soaked into a PPARγ apo-crystal by adding 0.5 µL TCBPA at a concentration of 1 mM suspended in well solution directly to the crystal drop. The crystals were soaked for 4 days. For co-crystals of PPARγ in complex with TBBPA, 1 µL protein solution was mixed with 1 µL well solution containing 1 M trisodium citrate, 100 mM HEPES, pH 7.5, 3.5% 1,2-propanediol, and 0.2 mM TBBPA ligand, for a molar ratio of 1:2 of protein:ligand in the drop. Crystals appeared after 1 day and grew to about 200 µm within a few days. Crystals were transferred to a cryoprotectant (well solution containing 20% glycerol and the corresponding ligand at a concentration of 1 mM) and frozen in liquid nitrogen.

*Crystallographic data collection, processing, and structure refinement.* We collected diffraction data at the ID14-1 beamline at 2.55 Å and 2.70 Å resolution for TBBPA-PPARγ and TCBPA-PPARγ complexes, respectively, using an ADSC Quantum Q210 CCD detector at the European Synchrotron Radiation facility (ESRF, Grenoble, France). Diffraction data were processed using MOSFLM (Leslie 2006) and scaled with SCALA from the CCP4 program suite (Collaborative Computational Project 1994). Structures were solved by using the previously reported structure 2ZVT (Waku et al. 2009) from which the ligand was omitted. Initial F_o_-F_c_ difference maps showed significant signals for the ligand, which could be fitted accurately into the electron density. The structures were modeled with COOT (Emsley and Cowtan 2004) and refined with phenix.refine from the PHENIX program suite (Afonine et al. 2005).

## Results

**Figure 2:**
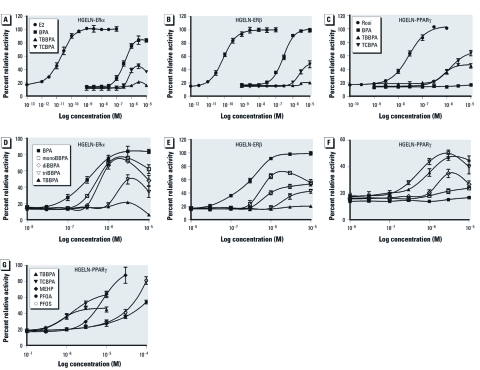
Results of luciferase assays showing dose–response curves for BPA and its halogenated analogs (TBBPA and/or TCBPA; *A–G*), and lower brominated analogs (monoBBPA, diBBPA, and triBBPA; *D–F*), as well as MEHP, PFOA, and PFOS (*G*), in HGELN‑ERα (*A,D*), HGELN‑ERβ (*B,E*), and HGELN‑PPARγ (*C,F,G*) cells. Results are expressed as a percentage of luciferase activity measured per well (mean ± SEM; *n* = 4) relative to the value obtained with 10 nM E2 (HGELN‑ERα and ERβ; *A,B,D,E*) and 100 nM rosiglitazone (Rosi; HGELN‑PPARγ; *C,F,G*).

*Halogenated BPA derivatives activate human ER*α, *ER*β, *and PPAR*γ. We monitored the agonistic potential of BPA and halogenated derivatives using stably transfected HGELN-ERα, -ERβ, -PPARα, -PPARδ, and -PPARγ cell lines, allowing for a comparison of the effect of compounds on human ER and PPAR subtypes in a similar cellular context. All compounds were first tested on the HGELN parental cell line containing only the reporter gene. We observed some toxicity at ligand concentrations of ≥ 10 μM but no unspecific modulation of luciferase expression (data not shown). We then characterized the activity of BPA, TCBPA, and TBBPA on HGELN-ER cell lines. As shown in Figure 2A and B, despite a reduced affinity relative to E2, BPA exerted an almost full agonistic activity toward both ERα and ERβ. In contrast, TBBPA had little effect on either ER, whereas TCBPA partially activated both receptor subtypes with a slight preference for ERα. Similar experiments carried out using HGELN-PPAR cells demonstrated that none of the compounds tested notably affected PPARα or PPARδ activity (data not shown). In contrast, both TBBPA and TCBPA were capable of partially activating PPARγ, despite being approximately 100-fold less potent than the reference pharmaceutical compound rosiglitazone (Figure 2C). The parent compound BPA failed to activate PPARγ. The occurrence of lower-brominated BPA analogs in the environment prompted us to measure their activity in HGELN-ER cell lines and HGELN-PPARγ cells (Figure 2D–F). Figure 2D shows that all brominated BPA congeners were partial ERα agonists with graded activities. BPA, monoBBPA, and diBBPA displayed the highest transactivation efficiency followed by triBBPA, whereas TBBPA had almost no activity in the HGELN-ERα cells. These compounds were also tested in the HGELN-ERβ cell line, providing a similar partial activity and ranking order of estrogenic potency (Figure 2E). MonoBBPA and diBBPA, the brominated analogs characterized by the highest estrogenic potency but the lowest molecular weight, exhibited a slight ERα selectivity. Interestingly, when assayed in HGELN-PPARγ cells, the halogenated compounds ranked in the reverse order, with triBBPA and TBBPA showing the highest potency to induce luciferase gene expression, followed by diBBPA and monoBBPA (Figure 2F). Finally, we compared TBBPA and TCBPA with the well-known environmental PPARγ ligands MEHP (Feige et al. 2007), PFOS, and PFOA (Takacs and Abbott 2007) [see Supplemental Material, Figure 1 (http://dx.doi.org/10.1289/ehp.1003328)]. As shown in Figure 2G, the halogenated BPAs triggered PPARγ activation at 10- to 100-fold lower concentrations than the other candidate PPARγ disruptors.

**Figure 3:**
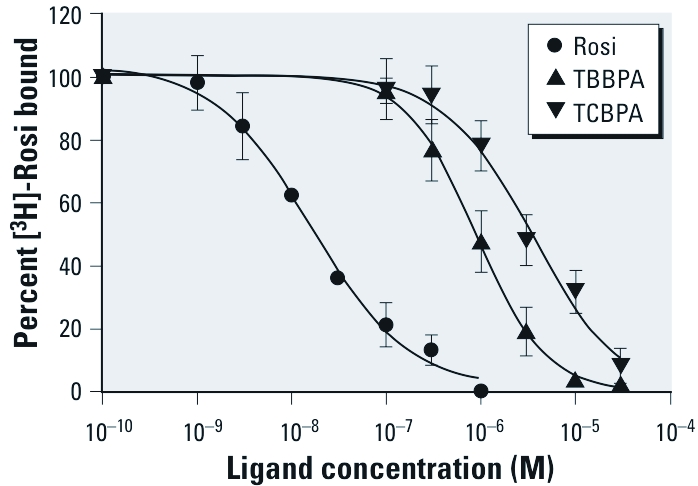
Competitive inhibition of [^3^H]-rosiglitazone (Rosi) binding in HGELN‑PPARγ cells incubated with different concentrations (0.001–30 μM) of Rosi, TBBPA, and TCBPA in the presence of 3 nM [^3^H]‑Rosi. Values are the mean ± SD from four separate experiments.

*Binding activity of TBBPA and TCBPA to human PPAR*γ *receptor.* To further characterize the interaction between human PPARγ and halogenated BPA derivatives, we performed whole-cell competitive binding assays using HGELN-PPARγ cells. TBBPA and TCBPA competitively inhibited the binding of [^3^H]-rosiglitazone to PPARγ (Figure 3). The half maximal inhibitory concentration (IC_50_) values for rosiglitazone, TBBPA, and TCBPA were 12.0 nM, 0.7 μM, and 6.0 μM, respectively. Together with transactivation assays (Figure 2C,F,G), these data demonstrate that TBBPA and TCBPA bind to human PPARγ and activate the receptor at concentrations in the micromolar range.

**Figure 4:**
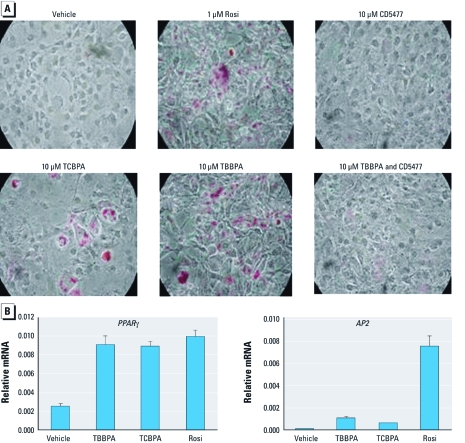
TBBPA and TCBPA induce adipogenesis through PPARγ. Two-day postconfluent 3T3L1 cells were treated for 8 days with 10 μg/mL insulin with vehicle (0.1% DMSO) or the ligands as indicated. Rosi, rosiglitazone. (*A*) Entire wells imaged after Oil Red O staining. (*B*) Quantitative real-time PCR of *PPAR*γ and adipocyte-specific fatty acid–binding protein *AP2 *expression levels in postconfluent 3T3-L1 cells treated with the ligands for 24 hr. Data were normalized to 18S or 36B4 controls and plotted as average fold induction ± SE (*n* = 3 per treatment).

*Halogenated BPAs promote adipocyte differentiation through PPAR*γ. Having shown that halogenated BPAs are PPARγ ligands, we investigated the action of TBBPA and TCBPA on endogenous genes by studying their ability to induce adipogenesis, a well-characterized PPARγ-regulated function. As we expected, treatment of 3T3L1 preadipocytes with the full PPARγ agonist rosiglitazone strongly induced adipogenesis, as evidenced by Oil Red O staining, whereas the PPARγ antagonist CD5477 (le Maire et al. 2009) did not induce adipocyte differentiation (Figure 4A). TCBPA and TBBPA at 10 μM also induced adipogenesis, whereas co-treatment with CD5477 inhibited the adipogenic action of TBBPA, indicating that halogenated BPAs mediate adipogenesis via PPARγ. Adipocyte differentiation by TBBPA and TCBPA was further confirmed by examining the endogenous expression of two PPARγ target genes, namely *ApoA2/FABP4* (*AP2*) and *PPAR*γ itself (Figure 4B). Whereas *PPAR*γ was expressed at similar levels upon treatment with halogenated BPAs or rosiglitazone, *AP2* was expressed to a much lesser extent after treatment with either TBBPA or TCBPA compared with rosiglitazone. This differential expression level of the two genes could reflect partial agonism of halogenated BPAs.

**Figure 5:**
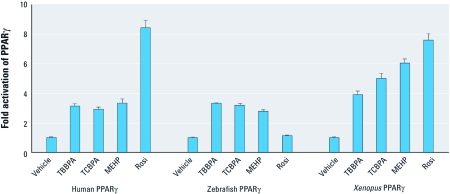
Effect of halogenated BPAs on activation of human, zebrafish, and *Xenopus* PPARγ. HeLa cells transiently transfected with (GALRE)5-βglobin-luciferase and pSG5-GAL4-PPARγ (human and zebrafish) or (PPRE)3-TK-luciferase and pSG5-PPARγ (*Xenopus laevis*) plasmids were incubated with 10 μM TBBPA, 10 μM TCBPA, 10 μM MEHP, or 1 μM rosiglitazone (Rosi) to assess their agonist potential on PPARγ. Values are the mean ± SD of three separate experiments.

*TBBPA and TCBPA are activators of zebrafish and* Xenopus PPARγ. Because the amino acid sequence of PPARγ differs between mammals and other species, we carried out transient transactivation assays to examine the ability of TBBPA and TCBPA to act as agonists of zebrafish and *Xenopus* PPARγ (Figure 5). These two animal species are often used as *in vivo* models to evaluate the impact of environmental compounds on organisms (Fini et al. 2007; Legler et al. 2002). This experiment confirmed that TBBPA, TCBPA, and MEHP are all activators of human PPARγ. We also found that TBBPA and TCBPA activated zebrafish *PPAR*γ, whereas MEHP appeared to be a slightly weaker ligand, and rosiglitazone was completely inactive. In contrast, all compounds, including rosiglitazone, activated *Xenopus* PPARγ. Together, our data indicate that halogenated BPA can disrupt the activity of PPARγ from different species.

**Figure 6:**
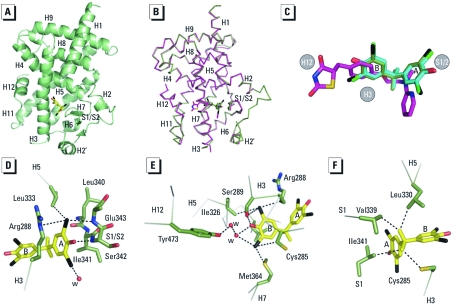
Crystal structures of PPARγ LBD in complex with TBBPA and TCBPA. (*A*) Overall structure of the TBBPA-bound PPARγ LBD. The polypeptide backbone is illustrated as a green ribbon, and TBBPA is shown in stick representation with each atom type: carbon, yellow; oxygen, red; bromine, black. (*B*) Superimposition of the co-crystal structure of TBBPA-bound PPARγ LBD on the structure with rosiglitazone (PDB code 2PRG; Nolte et al. 1998). Carbon atoms are shown in green in the TBBPA complex and in magenta in the rosiglitazone structure. (*C*) TBBPA (carbon, green; oxygen, red; bromine, black), TCBPA (carbon, cyan; oxygen, red; chlorine, green), and rosiglitazone (carbon, magenta; oxygen, red; nitrogen, blue; sulfur, yellow) as they appear in their respective LBP. (*D,E,F*) PPARγ residues in contact with TBBPA cycle A (*D*), cycle B (*E*), and the linker region (*F*). Key interactions are highlighted as black dashed lines; water molecules are shown as red spheres.

*Structural analysis of the TBBPA- and TCBPA-PPAR*γ *complexes.* Finally, we solved the crystal structures of TBBPA and TCBPA bound to the PPARγ LBD to reveal the mechanism by which these compounds, which are structurally unrelated to known PPARγ ligands, bind to and activate this receptor [see Supplemental Material, Table 1 (http://dx.doi.org/10.1289/ehp.1003328)]. As exemplified by the PPARγ–TBBPA complex (Figure 6A), the structures reveal the canonical tertiary fold of agonist-bound nuclear hormone receptor LBDs (Bourguet et al. 2000). The TBBPA and TCBPA complex structures are indistinguishable, with a root mean square deviation (RMSD) value of 0.29 Å for superimposed alpha carbons (see Supplemental Material, Figure 2) and nearly identical to that of PPARγ in complex with the agonist rosiglitazone (Nolte et al. 1998), with an RMSD value of 0.62 Å (Figure 6B). Omission of F_o_-F_c_ difference maps for the TBBPA and TCBPA structures revealed clear density for the ligands that could be positioned unambiguously into the PPARγ ligand-binding pocket (LBP) (see Supplemental Material, Figure 3B). PPARγ displays a large LBP that extends from the C-terminal helix H12 to the β-sheet S1/S2 so that halogenated BPA occupies a small portion of the LBP (Figure 6A). Whereas rosiglitazone occupies a region of the LBP spanning H11/H12 to the β-sheet S1/S2, TBBPA and TCBPA occupy only the region between H3 and the β-sheet S1/S2, with cycle A nestled between H3 and the β-sheet (Figure 6B,C). In contrast with rosiglitazone, a consequence of the smaller size of halogenated BPAs is that they do not interact directly with H12 (Figure 6B,C). A close look at the LBP shows that the phenol groups of the BPA derivatives are involved in hydrogen bonds. The hydroxyl group from cycle A (Figure 6D) interacts with the main chain nitrogen atom of Ser342 (β-sheet S1/S2), whereas the second one (cycle B) is hydrogen-bonded to Ser289 in H3 and Tyr473 from H12 through a water-mediated hydrogen bond network (Figure 6E). TBBPA and TCBPA contain four halogen atoms that contribute to ligand binding through van der Waals interactions (Figure 6D,E). Additional interactions involving the ligand backbone were also observed (Figure 6F). Interestingly, comparison of human, mouse, and zebrafish PPARγ sequences reveals several residue differences, which could explain the differential ligand specificity of the various species (see Supplemental Material, Figure 3A). In particular, the replacement of human PPARγ Gly284 and Cys285 by serine and tyrosine residues in zebrafish PPARγ provides a rationale for the weak binding affinity of rosiglitazone for this receptor compared with that observed for the human homolog (see Supplemental Material, Figure 3B). In contrast, the different binding mode of halogenated compounds allows both human PPARγ and zebrafish PPARγ to accommodate TBBPA and TCBPA (see Supplemental Material, Figure 3B).

## Discussion

Mounting data indicating the presence of halogenated bisphenols in environmental and human samples clearly suggest that these compounds should be considered an emerging class of contaminants whose cellular targets and effects require better understanding (Cariou et al. 2008; de Wit et al. 2010; Fernandez et al. 2007; Guerra et al. 2010). Brominated BPA analogs result mainly from the extensive use of TBBPA as a flame retardant, whereas a growing body of evidence suggests that chlorinated BPA analogs arise from the abiotic chlorination of BPA residues (Fukazawa et al. 2001). In the present study, we investigated the possible role of BPA and halogenated BPA derivatives as environmental ligands for ERs and PPARs.

We used HeLa cells, which are characterized by low intrinsic metabolic capabilities, to examine ER and PPAR activities to minimize metabolic biotransformations of tested compounds, which potentially can lead to a misinterpretation of *in vitro* results. Toxicity of halogenated BPA derivatives toward HeLa cell lines was observed at concentrations > 10 µM, which is fully consistent with previous reports showing that both TBBPA and TCBPA are more toxic than BPA (Nakagawa et al. 2007). For brominated analogs, the ranking order of estrogenic potency in HELN-ERα and HELN-ERβ cell lines was monoBBPA > diBBPA > triBBPA, whereas TBBPA showed no estrogenic activity at all. In a similar study using T47D breast cancer cells, Meerts et al. (2001) reported similar findings. Interestingly, in HGELN-PPARγ cells, the ability of BPA analogs to activate PPARγ was reversed, with TBBPA = triBBPA > diBBPA >>> monoBBPA. The activity of TCBPA was similar to that of TBBPA.

Full agonists of PPARγ (e.g., rosiglitazone) have been reported to fully activate their cognate receptor by directly interacting with and stabilizing helix H12, whereas compounds that do not directly contact H12 behave as weak or partial agonists by stabilizing other regions of the LBD, including H3 and the β-sheet S1/S2 (Bruning et al. 2007; Kallenberger et al. 2003). Both the functional and structural studies reported here support this model of partial PPARγ agonism by TBBPA and TCBPA. The rather weak affinity of halogenated BPAs for PPARγ compared with rosiglitazone can be explained by their smaller size and correspondingly fewer direct atomic contacts with the protein. Notably, whereas rosiglitazone is engaged in five hydrogen bonds with the protein, only two hydrogen bonds are observed between TBBPA/TCBPA and PPARγ [see Supplemental Material, Figure 4 (http://dx.doi.org/10.1289/ehp.1003328)]. The remaining contacts involve 89, 79, and 75 van der Waals interactions for rosiglitazone, TBBPA, and TCBPA, respectively (with a distance cutoff of 4.20 Å). The combination of significantly smaller size and the loss of stabilizing interactions between the halogen atoms of the halogenated BPA derivatives and PPARγ most likely accounts for the absence of significant interaction between the parent compound BPA and this receptor. Conversely, whereas the large LBP of PPARγ (Nolte et al. 1998) can readily accommodate the addition of bulky bromine or chlorine atoms, the much smaller LBP of the ERs cannot, thus providing an explanation for the differential pattern of interactions of halogenated BPAs with the two receptor types. It is noteworthy that some halogenated BPAs, including TCBPA and diBBPA, can interact with both ERs and PPARγ. This dual activity could increase the toxicity of these compounds compared with BPA or TBBPA, which are ER- and PPARγ-selective ligands, respectively. The comparison of the adverse effects induced by the two types of compounds through *in vivo* experiments should provide information on whether the dual ER/PPAR halogenated-BPA ligands display a higher EDC potency on reproductive and metabolic functions than more selective congeners. Until now, few environmental compounds (including MEHP, PFOS, PFOA, and organotins) have been found to interfere significantly with PPARγ signaling (Feige et al. 2007; Grün and Blumberg 2006; Grün et al. 2006; Takacs and Abbott 2007). In this regard, we recently reported that organotins potently activate RXR/PPARγ heterodimers essentially through binding to the RXR subunit (le Maire et al. 2009). Conversely, the functional and structural data presented here demonstrate that halogenated BPAs are capable of activating PPARγ via direct interactions characterized by binding affinities that are 10- to 100-times higher than other proposed PPARγ disruptors.

The discovery of a novel chemical class of PPARγ activators strengthens the hypothesis that environmental ligands could be involved in the disruption of energy balance in humans and wildlife. As with other EDCs, perinatal exposure could play a critical role. According to Cariou et al. (2008), significant levels of TBBPA can be found in human cord blood (200 pg/g fresh weight) and maternal milk (0.1–37.4 ng/g lipid weight), demonstrating both prenatal and postnatal exposure in a large fraction of the population. Furthermore, as other RXR (the main active form of PPARγ is the RXR/PPARγ heterodimer) and PPARγ activators are also present in the environment, additive (acting only through PPARγ) and synergistic (acting through both RXR and PPARγ) effects could occur and further increase the risk of metabolic diseases. In this regard, the “cocktail effect” resulting from a concomitant exposure to organotins and halogenated-BPAs could be particularly deleterious.

## Supplemental Material

(660 KB) PDF
