# Supplementary material for: Peroxisome Proliferator-Activated Receptor γ Is a Target for Halogenated Analogs of Bisphenol A
Source: Environ Health Perspect. 2011 May 11;119(9):1227–32. doi: 10.1289/ehp.1003328 (PMC3230400; doi:10.1289/ehp.1003328)
Supplement: (660 KB) PDF [file ehp.1003328.s001.pdf]

Supplemental Material

Peroxysome Proliferator-Activated Receptor γ is a Target for Halogenated Analogues of Bisphenol-A.

Anne Riu[1], Marina Grimaldi[2,3,4,5], Albane le Maire[6,7], Gilbert Bey[8], Kevin Phillips[9], Abelhay Boulahtouf[2,3,4,5], Elisabeth Perdu[1], Daniel Zalko[1], William Bourguet[6,7] and Patrick Balaguer[2,3,4,5*]

[1]INRA, UMR 1089 Xénobiotiques, 31027 Toulouse Cedex 3, France ; [2]IRCM, Institut de Recherche en Cancérologie de Montpellier, Montpellier, F-34298, France; [3]INSERM, U896, Montpellier, F-34298, France ; [4]Université Montpellier 1, Montpellier, F-34298, France; [5]CRLC Val d'Aurelle Paul Lamarque, Montpellier, F-34298, France ; [6]INSERM U1054, Centre de Biochimie Structurale, Montpellier, France ; [7]CNRS UMR5048, Universités Montpellier 1 & 2, Montpellier, France ; [8]NovAliX, 67400 Illkirch, France ; [9]Methodist Hospital Research Institute, Houston, TX 77030

Table of Contents

Supplemental Table 1.

Data collection and refinement statistics p. 3

Supplemental Figure 1.

Chemical structures of MEHP, PFOA and PFOS. p. 4

Supplemental Figure 2.

TBBPA and TCBPA in the PPARg ligand-binding pocket. p. 5

Supplemental Figure 3.

Differences between human and zebrafish PPARγ ligand-binding pockets. p. 6

Supplemental Figure 4.

Comparison of the binding pockets in PPARγ/ligand complexes. p.7

Supplemental Table 1. Data collection and refinement statistics

**Complex**	TBBPA	TCBPA
**PDB code**	3OSW	3OSI
Space group	*C2*	*C2*
Cell dimensions a, b, c (Å)	93.09, 61.69, 118.46	92.93, 61.74, 118.64
β (deg)	102.75	102.79
Resolution range (Å)	48.97-2.55 (2.69-2.55)*	44.70-2.70 (2.85-2.70)*
No. of reflections	20873	18014
R_{sym}	0.068 (0.435)*	0.070 (0.345)*
I/sI	9.5 (2.1)*	9.3 (2.7)*
Completeness, %	96.6 (92.7)*	98.9 (99.1) *
Redundancy	2.9 (2.9)*	2.3 (2.3)*
**Refinement**		
Resolution range (Å)	45.40-2.55	44.70-2.70
R (%) / R_{free} (%)	23.4 /29.6	23.2/29.6
Number of atoms	4206	4145
No. protein atoms	4074	4028
No. ligand atoms	21	21
No. water molecules	106	91
Average *B*-factor (Å^2)		
Protein *B*-factor (Å^2)	47.63	48.62
Ligand *B*-factor (Å^2)	57.75	55.27
Water *B*-factor (Å^2)	43.72	38.98
Rmsd from ideality		
Bond lengths (Å)	0.008	0.008
Angles (°)	1.1	1.2
Ramachandran plot (%)		
Favored region	93.0	93.4
Additionally allowed regions	6.6	6.4
Generously allowed regions	0.4	0.2
Disallowed regions	0.0	0.0

*Values in parentheses are for highest resolution shell.

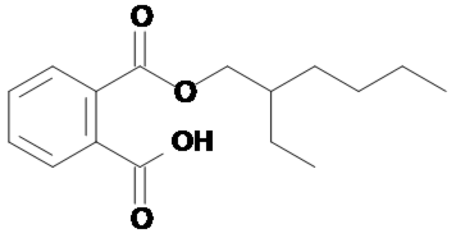

Mono-2ethylhexyl phthalate (MEHP)

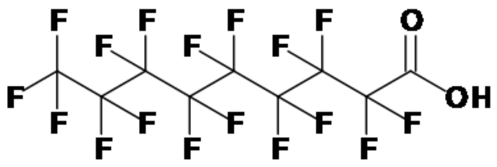

Perfluorooctanoic acid (PFOA)

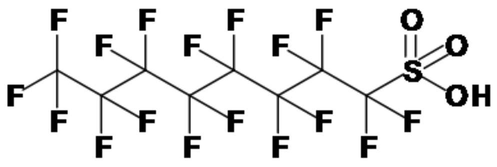

Perfluorooctanesulfonic acid (PFOS)

Supplemental Figure 1. Chemical structures of MEHP, PFOA and PFOS.

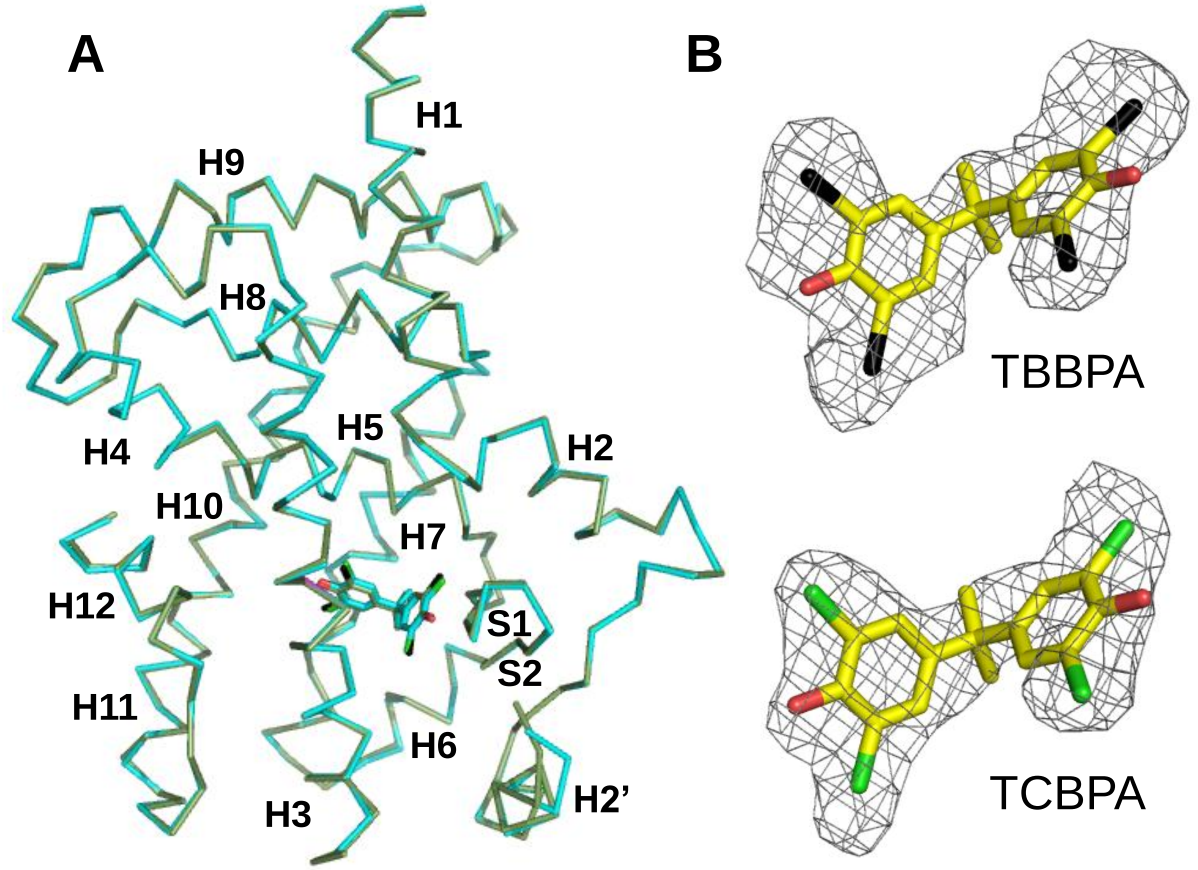

Supplemental Figure 2. TBBPA and TCBPA in the PPARγ ligand-binding pocket. (A) Carbon trace of superimposed PPARγ monomers bound to TBBPA (green) and TCPBA (cyan). (B) TBBPA and TCBPA in their respective Fo-Fc omit maps contoured at 2.5σ.

A

```
      275       285       295       305       315       325       335       345       355
            ** *** **  *                              *  **  *  *     ***      *              **
hPPAR KEVAIRIFQGCQFRSVEAVQEITEYAKSIPGFVNLDLNDQVTLLKYGVHEIIYTMLASLMNKDGVLISEGQGFMTREFLKSLRKPFGDFME
mPPAR KEVAIRIFQGCQFRSVEAVQEITEYAKNIPGFINLDLNDQVTLLKYGVHEIIYTMLASLMNKDGVLISEGQGFMTREFLKNLRKPFGDFME
zPPAR HEVELRFFHSYQSRSAEAISEVTEFAKSIPGFINLDLNDQVTLLKYGVIEVMIIMISPLMNKDGTLISYGQIFMTREFLKSLRKPFCEMME
```

B

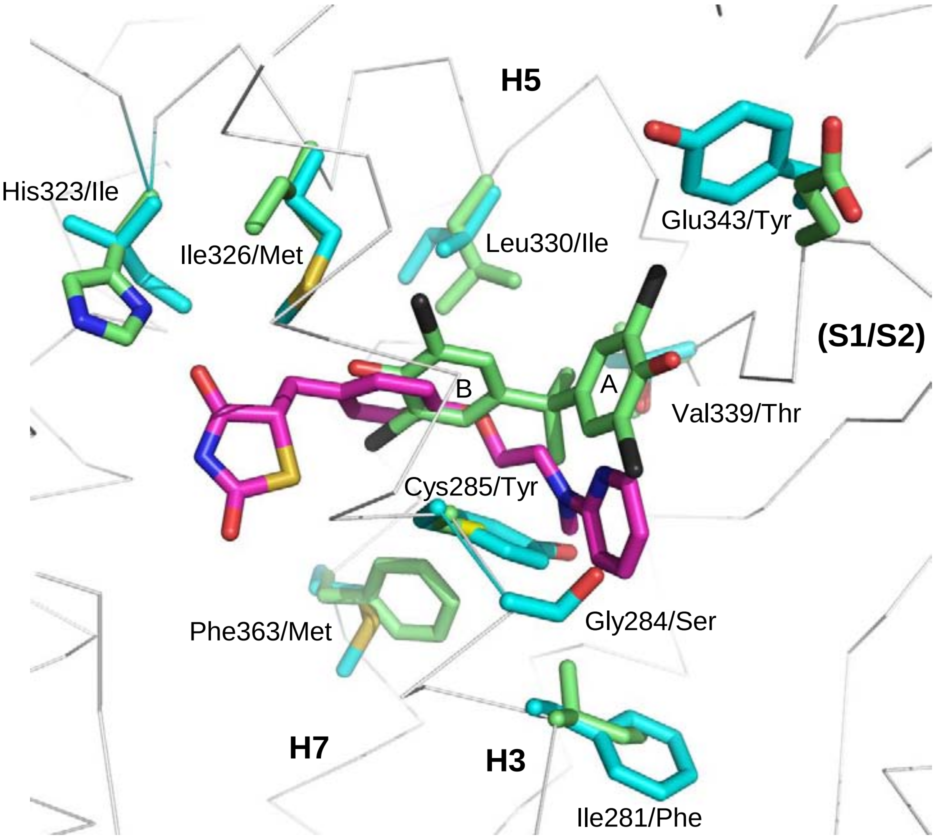

Supplemental Figure 3. Differences between human and zebrafish PPARγ ligand-binding pockets. (A) Sequence alignment of human, mouse and zebrafish PPARγ ligand binding pocket residues. Asterisks denote residues in contact with TBBPA and/or rosiglitazone (PDB code 2PRG). Interacting residues that differ between sequences are highlighted in red. (B) TBBPA (carbon atoms colored in green) and rosiglitazone (magenta, PDB code 2PRG) as they are positioned in the human PPARγ. Residues that differ in the ligand binding pocket of human and zebrafish PPARγ are displayed as green and cyan sticks, respectively.

A

Ligand	Contacts (total) (< 4.2 A)	Hydrogen bonds	van der Waals	IC50
Rosiglitazone	94	5	89	12.0 nM
TBBPA	81	2	79	0.7 μM
TCBPA	77	2	75	6.0 μM

B

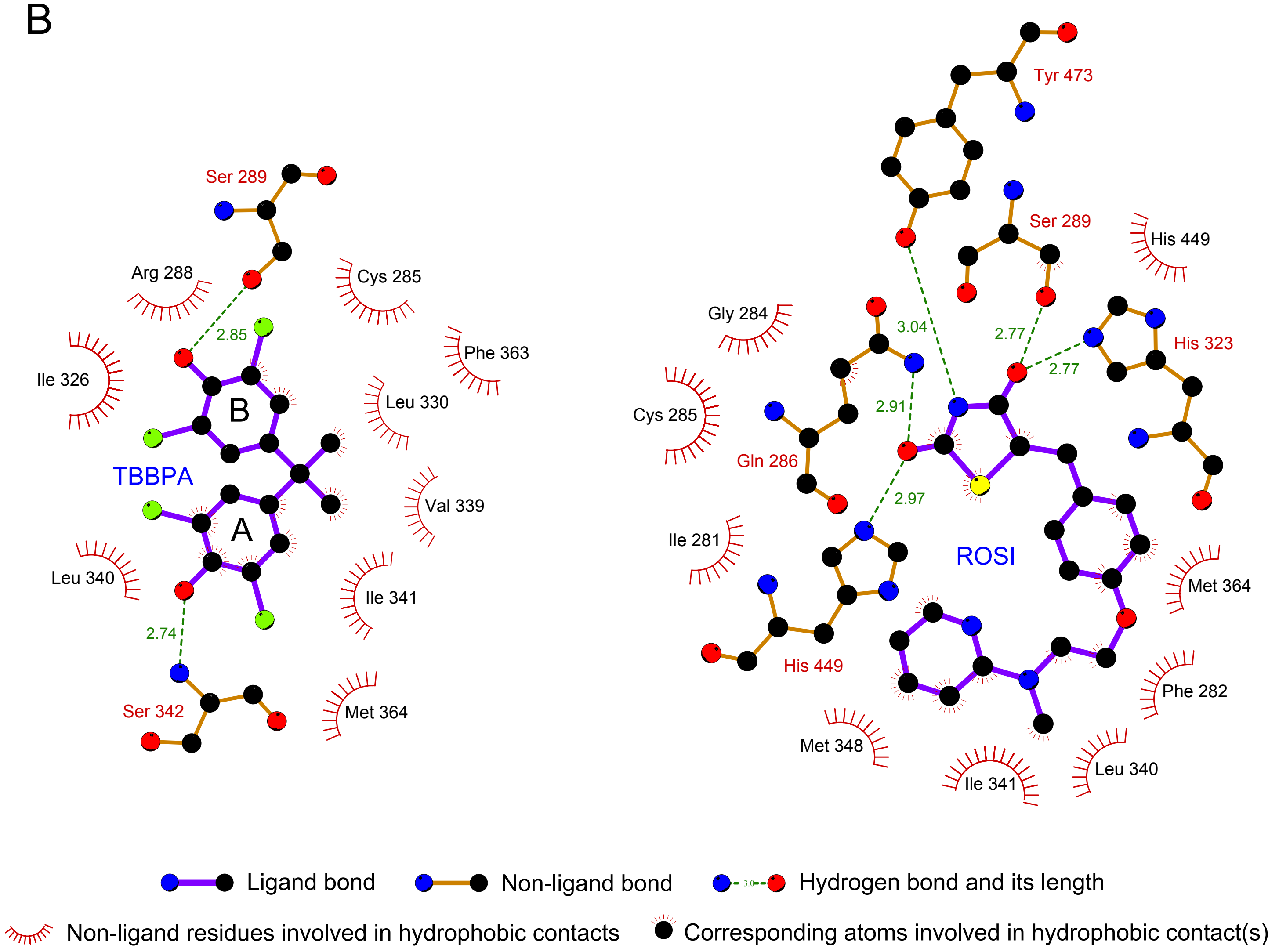

Supplemental Figure 4. Comparison of the binding pockets in PPARγ/ligand complexes. (A) Number of contacts (total, hydrogen bonds, van der Waals) and IC50 characterizing the complexes between PPARγ and rosiglitazone, TBBPA and TCBPA. (B) Schematinc drawing showing the interactions between PPARγ and the ligands in the TBBPA (left) and rosiglitazone (right) complexes.
